# New Position Candidate Identification via Clustering toward an Extensible On-Body Smartphone Localization System

**DOI:** 10.3390/s21041276

**Published:** 2021-02-11

**Authors:** Mitsuaki Saito, Kaori Fujinami

**Affiliations:** Department of Computer and Information Sciences, Tokyo University of Agriculture and Technology, 2-24-16 Naka-cho, Koganei, Tokyo 184-8588, Japan; perorokey@gmail.com

**Keywords:** clustering, context recognition, DBSCAN, machine learning, on-body device localization, extensible systems

## Abstract

On-body device position awareness plays an important role in providing smartphone-based services with high levels of usability and quality. Traditionally, the problem assumed that the positions that were supported by the system were fixed at the time of design. Thus, if a user stores his/her terminal into an unsupported position, the system forcibly classifies it into one of the supported positions. In contrast, we propose a framework to discover new positions that are not initially supported by the system, which adds them as recognition targets via labeling by a user and re-training on-the-fly. In this article, we focus on a component of identifying a set of samples that are derived from a single storing position, which we call new position candidate identification. Clustering is applied as a key component to prepare a reliable dataset for re-training and to reduce the user’s burden of labeling. Specifically, density-based spatial clustering of applications with noise (DBSCAN) is employed because it does not require the number of clusters in advance. We propose a method of finding an optimal value of a main parameter, Eps-neighborhood (*eps*), which affects the accuracy of the resultant clusters. Simulation-based experiments show that the proposed method performs as if the number of new positions were known in advance. Furthermore, we clarify the timing of performing the new position candidate identification process, in which we propose criteria for qualifying a cluster as the one comprising a new position.

## 1. Introduction

In carrying a smartphone, people often grasp the terminal by hand or store it in various positions, such as trouser pockets, jacket pockets, or bags. According to a survey on carrying a phone terminal, 17% of people decide where to store their terminals based on situational limitations [1], e.g., no pocket exists on a T-shirt and the terminal is too large for a trouser pocket to be comfortable for ongoing activities. These factors may fluctuate throughout the day, which leads to users changing the position throughout the day. Once an incoming call or text message is received and delivered to the user via vibration, the user might be successfully notified when the terminal is in their hands, while being missed in a trouser pocket. In addition, a wide variety of embedded sensors or sensor extensions allow for the system to extract the context of the user, e.g., engaging activity, person’s location, identity of pedestrian, and environmental conditions around the user. This enables the system to provide appropriate information/services to the user based on the context; however, the literature shows that the storing position of the terminal also affects the quality of the extracted context and, thus, the information/services of the system. This implies that the position of the terminal needs to be handled appropriately in each application to provide a designated quality for sensor-dependent services [2], in order to facilitate human–human communication [3], etc., in any storing position, which is often called the “on-body device localization” problem.

In the last 15 years, the problem has been addressed by a number of researchers using machine learning techniques, in which inertial sensors are often utilized due to the fact that the moving patterns of the terminal differ by storing positions and recognition features; additionally, recognition models have been investigated [4,5,6]. However, is such investigations, it was assumed that the positions to be recognized, i.e., classes, are fixed and that a recognition component classifies input data into one of predefined classes. Thus, data from unknown positions are assigned to an incorrect position anyway. For example, if the front and back pockets of trousers are considered as classification targets at the beginning, and the recognition model is trained, these two positions can be recognized; however, even if the user stores his/her terminal in a chest pocket, the recognition system classifies it into either a front or back pocket of trousers. This means that an application that uses positional information might not be able to work properly. A solution for addressing this issue is to support as many positions as possible; however, the burden of collecting data for training the classifier and the complexity of the classification task significantly increase, although the majority of users might use very few preferred positions.

Herein, we take a different approach, in which the positions that are supported by the system are adjusted to the user. An incremental position addition framework is proposed [7], which starts with a minimum set of popular positions implemented by the system’s developer and extends its supported position by detecting unknown positions on-the-fly, labeling with help from the user, i.e., the owner of the terminal, and retraining the classifier for the new position. A novelty detection technique is utilized to judge whether a sample to be classified is obtained from supported position or not. A sample judged as "not supported" is the candidate for a new class. In this article, we clarify the design principle of a component of identifying new position candidate. The decision on the identification of new classes is made against multiple instances of candidates, where more than two position candidates can be included. This is because people may store their terminals in more than two new positions in a particular period of time, e.g., a day, which requires the instances of candidates to be clustered prior to be labeled by the user.

The contributions of this article are summarized, as follows:

- A data-driven method that finds an optimal parameter value for clustering is heuristically developed, which does not need to specify the number of clusters in advance, yet it uses a dataset to train the position classification component. An evaluation shows that the proposed approach is comparable to existing methods that specify the number of clusters in advance and that estimate the number of clusters on-the-fly. The re-trained classifier performs well with high accuracy, i.e., an accuracy of more than 0.94.
- A condition that determines the time of performing a new position candidate identification process is presented as a result of experiments varying the number of samples and the breakdown of the samples of new class candidates. The estimated minimum time to collect a sufficient number of samples is appropriate, i.e., 5–12 min. in three datasets used in the evaluation, enough to be implemented in daily use.
- By integrating the design principles that are presented in this work with those already known in our previous work, a complete picture of the incremental position addition framework during walking is presented.

The remainder of the article is organized, as follows: Section 2 examines the work that is related to smartphone position recognition, and example applications of the machine learning technology used in this work are examined. The incremental position addition framework is presented with an introduction of the design principles already obtained in our previous work, as well as the research questions shown in Section 3. Section 4 proposes a method for finding optimal parameter values for clustering in new position candidate identification, which is followed by experiments for designing the timing of performing the identification process in Section 5. Finally, Section 6 concludes the article with future work.

## 2. Related Work

### 2.1. Usefulness of On-Body Smartphone Position Recognition

The position recognition of smartphones has shown considerable usefulness, in that it provides users with more useful ways to use a smartphone. First is the automatic selection of notification methods. One of the major functions of smartphones is to notify users of notifications, such as calls, e-mails, and coupon information. Possible notification methods include device vibration, ringtone, and light. Exler et al. [3] showed that there are appropriate notification methods, depending on the possession position from the perspective of the user’s awareness and preference. The work indicated that the recognizability of the three possession positions and the three notification methods varies, depending on the possession position and notification method. The appropriate notification method can be automatically selected if the smartphone itself can recognize the possession position.

Second is the realization of wearable sensing that takes the storing or carrying position of a smartphone into account. Wearable sensing refers to measuring the state of the surrounding environment of the user using devices that people wear on a daily basis [8,9]. Measurements are performed while using inertial sensors, such as acceleration and angular velocity sensors. built into the device, or using input devices, such as cameras and microphones. Wearable sensing has been extensively explored in recent years, because it is useful for users who wear the device to collect useful information without burdening or paying attention to the measurement. In particular, smartphone usage is widespread, even when going out, so they are effective as sensing devices. However, there is a problem, in that the acquired sensor value fluctuates, depending on the position of possession. Fujinami [2] showed that the values of the temperature and humidity sensors built into mobile devices change depending on the three positions of said mobile devices. Therefore, if the storing position of a mobile device is recognized in advance, then the acquired sensor value can be corrected to the correct answer value, which is, the sensor value at the neck, which can be useful for accurate environmental sensing.

Activity recognition using a smartphone aims to recognize a user’s behavior using the built-in inertial sensor. The target activities range from work that recognizes daily movements, such as walking and running, to work that recognizes focused movements, such as falling movements [10,11,12]. However, activity recognition has the problem that recognition accuracy decreases, depending on the position of the smartphone on the body [13]. In other words, the activity recognition accuracy when stored or carried in an unexpected position is considered to be significantly lower than when in the position assumed by the designer of the activity recognition system. One of the solutions to this problem is that the possession position of the smartphone is recognized in advance and, during activity recognition, the data used for the activity recognition and the recognition method are changed accordingly. Alanezi et al. [14] and Sztyler et al. [15] showed that activity recognition is performed with high accuracy by this approach when possessed in various positions.

### 2.2. On-Body Device Position Recognition

Many researchers have investigated smartphone position recognition because of the usefulness that is indicated in the previous section. Table 1 summarizes the storing positions of mobile devices found in the literature, which includes trouser pockets, chest pockets, shoulder bags, handbags, and hands. Here, the accuracy generally cannot be compared, because the experimental conditions and verification methods differ in each work. However, they all focus on recognizing the position of the device in one of the predefined positions. Hence, the system misrecognizes data from an unknown position as one of the known positions. Once the system knows that the data are obtained from an unknown position, it can take appropriate action, such as discarding the result and asking the user to label the data for registering the detected position to recognition targets.

### 2.3. Application of Machine Learning Techniques to Inertial Sensor Signals

In this work, a mobile device possession position recognition system was designed based on machine learning techniques, including pattern recognition, novelty detection, and clustering [31,32,33]. The existing work applied to inertial sensor values are presented after a brief description of the mechanism of each technique.

Regarding pattern recognition, a recognition model was generated using data with labels of *n* classes $A_{1}$ to label $A_{n}$ as training data. When test data are given to this model, it recognizes which of the labels ($A_{1}$ to $A_{n}$) it belongs to. During device position recognition, this approach was used with positions as labels [4,5,6]. After generating a recognition model from various position data, this work verified whether the position of the test data can be correctly recognized. Similarly, recognition technology was also used for activity recognition [10,11,12].

For novelty detection, a model was generated using data with *n* labels $A_{1}$ to label $A_{n}$ as training data. When test data are given to this model, a judgement is made on whether the data are known or unknown, which is, whether they belong to one of the classes $A_{1}$ to $A_{n}$. Yin et al. [34] treated five activities, such as walking and running as known activities, and all other activities as unknown activities. Guo et al. [35] and Yang et al. [36] performed novelty detection by changing the combination of known and unknown activities. Our work is related to these works in terms of novelty detection using the inertial sensors of mobile devices; however, our work treated the position of smartphones as a target.

Clustering identifies clusters by grouping samples that are expected to have the same label for multiple data with unknown labels, which belongs to unsupervised machine learning. THe application of clustering in inertial sensor signals reduces the burden of labeling the training data [37,38]. When generating a recognition model in activity recognition, it is necessary to label all the samples of the training data used; however, it is burdensome for humans to do this task manually. Therefore, by applying clustering to the training data and treating the samples in the same class as the same label, the burden of labeling can be significantly reduced. Clustering is used for the same purpose in our work; however, it is applied to data at unknown positions that are detected by novelty detection, rather than to the data that were initially collected as training data.

## 3. Incremental Position Addition Framework

### 3.1. Overview

**Figure 1:**
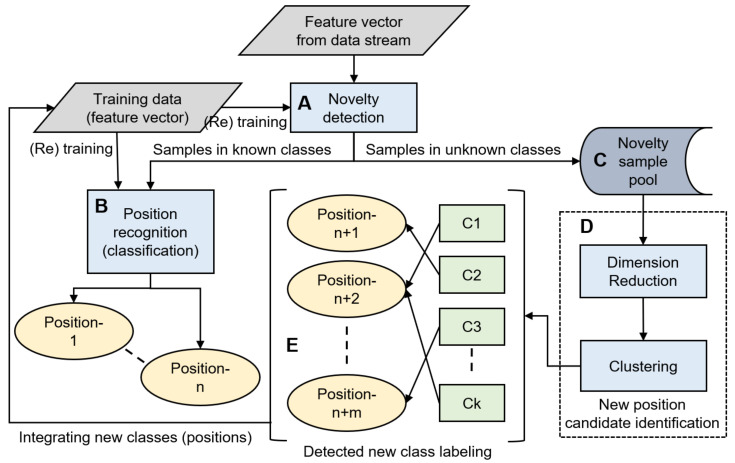
Framework of the incremental addition of smartphone carrying positions. (**A**) Novelty detection, (**B**) position recognition, (**C**) novelty sample pool, (**D**) new position candidate identification, and (**E**) detected new class labeling.

Our proposed framework consists of five major components: (A) Novelty detection, (B) position recognition, (C) novelty sample pool, (D) new position candidate identification, and (E) detected new class labeling (Figure 1) [7].

In the framework, multi-dimensional features are calculated against fixed-size windows in raw inertial sensor data streams. By applying the novelty detection technique, an evaluation is performed in order to determine whether each feature vector for position recognition can be classified into one of the *n* known positions (Figure 1A). If the feature vector is determined to belong to either of the known classes, it is forwarded to the position recognition component and classified as one of the *n* known positions (B). Otherwise, the vector is stored in the novelty sample pool (C). The saved samples are used to identify *k*-clusters of candidates for new positions (D) if a certain number of samples are collected or a certain time has elapsed. The role of clustering is to support the end users to label for new positions. In extreme cases, they can label sample by sample; however, this is burdensome. By contrast, only one labeling is enough if the samples in the novelty sample pool originate from one new position and forms a dedicated cluster. The identification of a new position candidate includes the following two functions: dimension reduction and clustering. Applying clustering in higher dimensions may produce incorrect outcomes; therefore, the dimension needs to be reduced before clustering. Clustering is adopted for samples using features that are converted to a low dimension by dimension reduction. Clusters are then labeled by the user him/herself for re-training the classifier. Some of the clusters can be merged into one class that is based on the decision of the user, and m(≤k)-new classes are generated accordingly (E). The position that a specific sample was derived from can be asked of the user. This labeling is not performed many times a day; in consideration of the burden on the user, it is performed approximately once a day, such as before going to sleep. Finally, the classifier of the position recognition is re-trained to support (n+m)-classes, given the newly labeled *m*-class data and those of existing *n*-known classes.

The class incremental learning without forgetting (CILF) is a state-of-the-art framework that detects unknown classes and adds them to new classes [39]. The CILF framework divides the apparent test data into windows, detects unknown classes, and then adds them to new classes for each window. However, at this time, there is no guarantee that a robust cluster can be created in the window. In contrast, in the proposed framework of this article, test data judged to be unknown are temporarily saved in the pool and then added to a new class after satisfying specific conditions. In other words, it is guaranteed that clustering can be performed after sufficient data have been accumulated to generate a cluster. Therefore, it is an advantage of our framework to collect an appropriate amount of data for constructing a new class and then adding it. Furthermore, in our proposed framework, whether the test data are known or unknown is judged at any time when the test sample appears. The data determined to be known are passed to the position recognition function, while the data determined to be unknown are stored in the pool in order to identify clusters in the later process. On the other hand, CILF simultaneously discovers known and unknown classes against input data in a relatively large window. Therefore, the novelty detection is not performed until specific amount of data for a window are accumulated in CILF. Because the position recognition component takes feature vectors that were calculated from known data as inputs in real time, the judgement on novelty (or non-novelty) should be made immediately once a feature vector is given to novelty detection component. For this reason, we consider it to be difficult for CILF to replace our proposed framework in applications that use the data of known class in a real time manner.

### 3.2. Our Previous Work and the Scope of This Article

In the design of a novelty detection component (A), we compared three popular novelty detection methods, i.e., one-class Support Vector Machine (OCSVM) [40], local outlier factor (LOF) [41], and isolation forest (IForest) [42], and we concluded that LOF is applicable here [7]. Furthermore, an ensemble novelty detection method is proposed, in which we showed that the accuracy of discrimination between the unknown and known positions was improved [43].

For new position candidate identification (D), an effective combination of existing dimension reduction and clustering methods was explored [44,45], in which principal component analysis (PCA) [46] and t-distributed stochastic neighbor embedding (t-SNE) [47] were considered as dimension reduction sub-components, while *k*-means [48], *X*-means [49], and density-based spatial clustering of applications with noise (DBSCAN) [50] were tested as the candidates of a clustering method. *k*-means is a popular clustering method that calculates the distance from the representative sample of each cluster to each sample and updates the cluster accordingly. The number of clusters needs to be specified at the time of calculation due to the nature of the algorithm. Meanwhile, *X*-means is a variation of *k*-means clustering, which internally applies *k*-means repeatedly by changing the number (*k*), and the one with the best performance is adopted. We also considered using the elbow method [51] as another method for finding the appropriate number of clusters; however, we concluded that it was inappropriate in this work, because it requires user involvement in determining the appropriate number of clusters.

**Figure 2:**
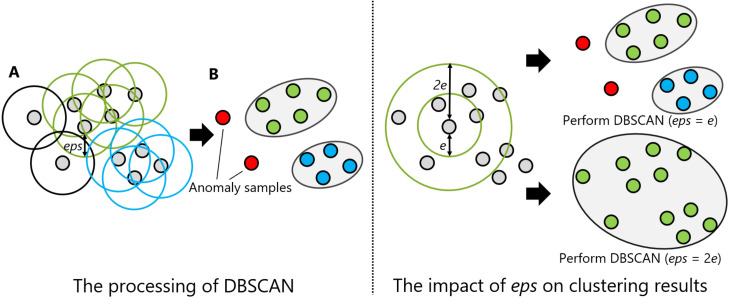
An illustration of density-based spatial clustering of applications with noise (DBSCAN): the left and right parts are the processing of DBSCAN and the impact of Eps-neighborhood (eps) on the clustering results, respectively.

**Figure 3:**
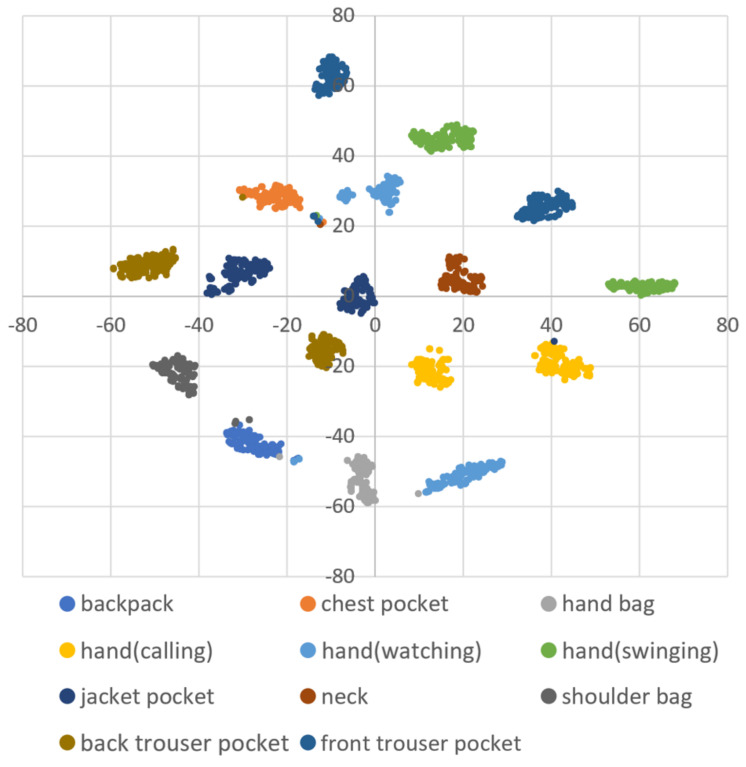
A two-dimensional visualization of the data of one subject using t-distributed stochastic neighbor embedding (t-SNE) in dataset A.

**Figure 4:**
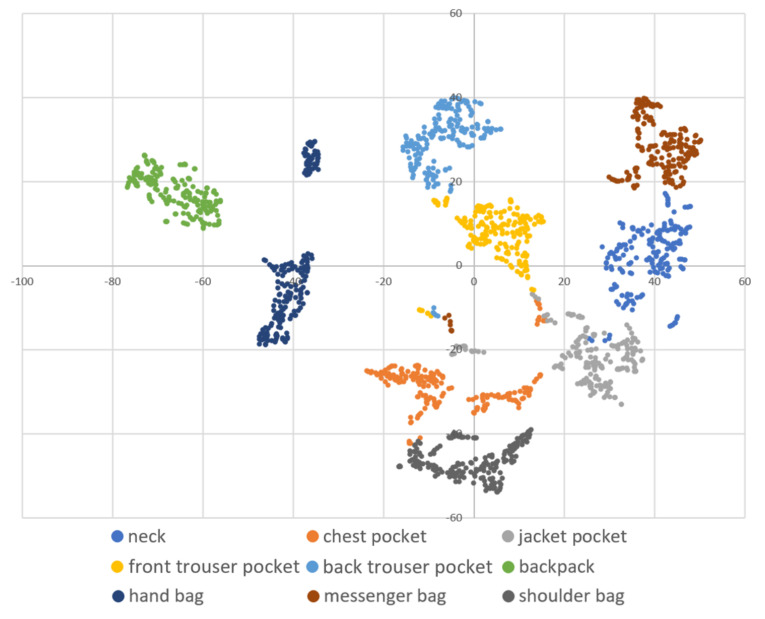
A two-dimensional visualization of the data of one subject using t-SNE in dataset B.

**Figure 5:**
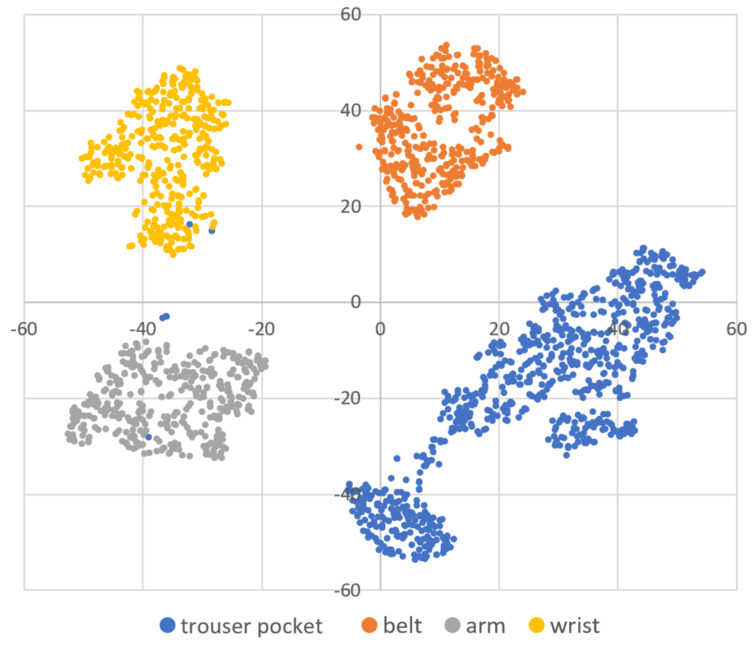
A two-dimensional visualization of the data of one subject using t-SNE in dataset C.

DBSCAN regards two samples as belonging to the same cluster when they are less than a certain distance apart and forms clusters. DBSCAN has a key parameter, called Eps-neighborhood (*eps*); it is also represented by ε. The left part of Figure 2 shows the processing of DBSCAN. For each sample of the given data, the other samples within a radius of *eps* are considered the same cluster (A). Subsequently, a sample with a small number of other samples within the radius of *eps* is judged to be an anomaly sample (B). A prominent characteristic of DBSCAN is that it can form clusters without specifying the number of clusters in the data, as opposed to *k*-means. Instead, *eps* is a very important parameter. As shown in the right part of Figure 2, the larger *eps*, the larger the distance between the two samples considered to be in the same cluster; however, too large a value of *eps* causes clusters to be merged, and the majority of samples belong to the same cluster. By contrast, if too small an *eps* is chosen, a large part of the data are not clustered and are denoted as outliers. This means that DBSCAN can be used to simultaneously detect anomalies with clustering. Figure 3, Figure 4 and Figure 5 show two-dimensional visualizations of the distributions of positions of one person in the three datasets used in this article (Section 4.2.3 provides details of these datasets). There are isolated samples at points away from some large clusters in each dataset, which are regarded as outliers.

Affinity propagation [52] and spectral clustering [53] are other methods that do not specify the number of clusters; however, they do not detect isolated samples. Additionally, when the number of samples given to the methods is *s*, the complexity of the clustering is $O(s^{2})$, $O(s^{3})$, and O(slog(s)) for affinity propagation, spectral clustering, and DBSCAN, respectively [52,54]. The clusters of positions are not always circular, as is prominent in dataset B. Spectral clustering and DBSCAN can handle such warped shape clusters; however, by taking into account the execution load when the proposed framework is operated in a smartphone, DBSCAN had advantages and, thus, was adopted as a method that does not specify the number of clusters. Additionally, hierarchical DBSCAN (HDBSCAN) is a modified method of DBSCAN [55], which performs clustering with high accuracy, even if the given data contain clusters of different densities. The density of the data in each dataset seems to be equal, regardless of the position, as shown in Figure 3, Figure 4 and Figure 5. Therefore, we considered that the application of the original DBSCAN would be sufficient.

Because of the fact that the system deals with time series data, data stream clustering [56] might seem appropriate for clustering techniques, rather than non-stream data clustering, such as *k*-means and DBSCAN. A typical use of data stream clustering is to understand the evolution of clusters over a time horizon by comparing clusters between two or more time points, e.g., are there new clusters or did original clusters disappear between time $t_{2}$ and $t_{1}$? In contrast, in the proposed framework, clusters are registered as positions that are supported by the position recognition component (Figure 1B) once they are identified as a result of clustering (Figure 1D), labeling by the user (Figure 1E), and retrained. This means that the role of the clustering is to identify unknown positions, unregistered positions in other words, in the most recently obtained data, and that the change of the set of device storing positions is of no interest to the system. Therefore, the non-stream data clustering method was adopted herein.

As a result of a comparison, t-SNE was shown to be effective for dimension reduction, regardless of the clustering method. In addition, DBSCAN achieved comparable or better performance than *k*-means and *X*-means, given optimal *eps*. Therefore, we concluded that DBSCAN is the most suitable clustering method for the new position candidate identification in the three methods, in that it does not require the user to make any input. In this article, we answered the following questions regarding the design principle of the new position candidate identification process that remain unexplored in previous works.

**Q1:**  How to specify the optimal value of *eps*? (Section 4)
**Q2:**  When to perform a new position candidate identification process? (Section 5)

## 4. Designing a New Position Candidate Identification Component

### 4.1. Finding an Optimal Value of eps

In this section, a method for finding an optimal value of *eps* in DBSCAN is proposed. As a result of evaluating *X*-means as a method for estimating the number of clusters in our previous work, this method is not capable of appropriately estimating the value, so this paper focused on finding the optimal *eps* in DBSCAN. On the contrary, there are other methods besides *X*-means that automatically calculate the number of clusters, such as Upper Tail method [57]. However, there are already learned labeled known classes in the proposed framework. Therefore, we thought that it would be beneficial to leverage data from known classes in the framework for clustering, because the clustering method can rely on the existing information, called labels, rather than using existing methods for calculating the number of clusters only from pooled data whose labels are still unknown. This was a motivation for proposing an optimal *eps* finding method.

#### 4.1.1. Parameters Used for Evaluating the Appropriateness of *eps*

The proposed framework assumes to have a pre-trained classifier before end users start while using the system, which indicates that labeled data for a fixed number of positions exist. In the proposed method, DBSCAN is performed on these data, and some evaluation parameters are calculated from the clustering results. Specifying an optimal value of *eps* is made based on these parameters.

First, we defined $R_{outlier}$ by Equation (1), where the number of samples determined as outliers and the number of samples given for clustering are $N_{outlier}$ and $N_{clustering}$, respectively. $R_{outlier}$ is the rate of samples judged to be anomalies. It is problematic if $R_{outlier}$ becomes too large, because the samples that are determined as outliers are removed and then it prevents clusters from forming properly.

(1) $$\begin{array}{c} R_{outlier}=\frac{N_{outlier}}{N_{clustering}} \end{array}$$

Second, we introduced parameter $D_{K}$ defined by Equation (2), given that the number of clusters identified by clustering is $K_{identified}$ and the ideal number of clusters is $K_{ideal}$. It is important in clustering that the number of identified clusters is close to the number of positions actually given. Here, there are positions that may be carried on the left and right sides of the body, such as trouser pockets. We considered that such positions should be clustered into two different clusters on the left and right. This is because, when users label a new position candidate, they can label with a high degree of freedom, such as giving different labels for left and right, or giving the same label and integrating them. Therefore, we defined an ideal number ($K_{ideal}$) that represents the number that should be identified by clustering. If the sample that is given to the clustering contains positions collected from the left and right, $K_{ideal}$ is added to the actual number of positions accordingly.

$D_{K}$ represents the difference between the number of identified clusters and $K_{ideal}$. The closer $D_{K}$ is to 0, the more precise the finding of the number of clusters. Moreover, as shown in our previous work, it is desirable that $D_{K}$ be positive rather than negative [45].

(2) $$\begin{array}{c} D_{K}=K_{identified}-K_{ideal} \end{array}$$

Finally, we used the Fowlkes–Mallows Index (FMI)[58] that is defined by Equation (3). FMI is calculated by comparing the clustering results and correct labels attached when collecting the experimental data, i.e., ground truth. Here, the sample pairs that belong to the same clusters in both the clustering results and the correct labels are regarded as the true-positive cases and are represented as TP, while FP indicates the number of sample pairs that belong to the same clusters in correct labels and not in the clustering results, i.e., the false-positive cases. Moreover, the number of sample pairs that belong to the same clusters in the clustering results and not in the correct labels is FN, representing the false-negative cases. For example, if Position-1 is the correct label for two samples that are clustered in the same cluster, TP increases by 1, but, if they are clustered in different clusters, FP increases by 1. Additionally, if Position-1 is the correct label for one sample and Position-2 is the correct label for another sample that are clustered in the same cluster, then FN increases by 1. FMI takes a value from 0.0 to 1.0, and it shows similarity between the clustering results and correct labels. Thus, a high FMI value is desirable.

(3) $$\begin{array}{c} FMI=\frac{TP}{\sqrt{\left(TP+FP)(TP+FN\right)}} \end{array}$$

#### 4.1.2. Finding an Optimal eps for Hyperparameter Tuning

**Figure 6:**
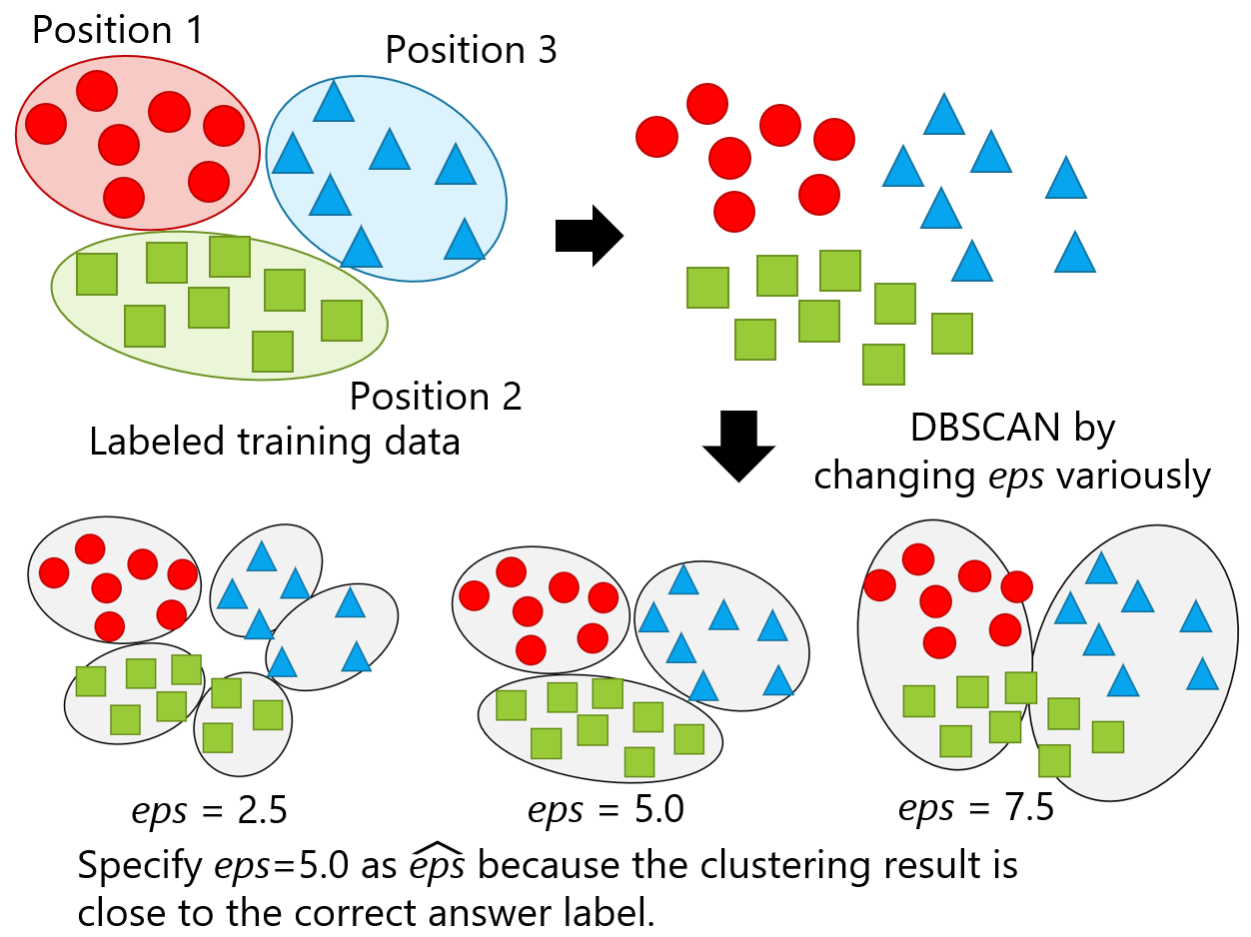
A simplified version of the proposed method with three levels of *eps*.

The proposed framework assumes to have a pre-trained classifier before end users start using the system, which indicates that labeled data for a fixed number of positions exist, as mentioned in the previous section. We also assumed that an optimal *eps* obtained by performing DBSCAN using another person’s data is compatible to that of a particular user. Thus, we leveraged the data from other persons to specify optimal *eps* for hyperparameter tuning before using the system. DBSCAN is performed on the labeled data while changing the value of *eps*. Subsequently, *eps* with the clustering result closest to a correct label is judged as an optimal value, represented as $\hat{eps}$. Figure 6 illustrates a simplified version of the proposed method with an example, in which *eps* assumes to be set to 2.5, 5.0, and 7.5, and the clustering result at *eps* = 5.0 is close to the clusters of correct labels, as seen in the upper left of the figure. Thus, $\hat{eps}$ was found to be 5.0.

**Figure 7:**
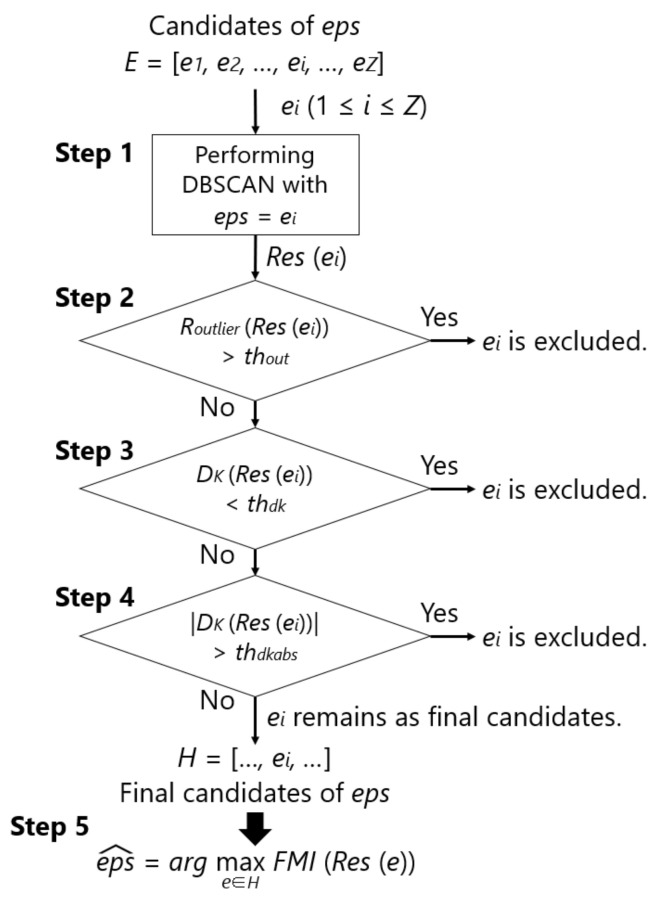
Flowchart of the $\hat{eps}$ search method.

The detailed parameter search consists of the following five steps. Figure 7 also shows the flowchart of this method. Here, a set of *eps* are given as candidates of $\hat{eps}$, which is denoted as *E*, and $e_{i\in [1,Z]}$ represents an element of *E*, with the number being *Z*. Through the five steps, one $\hat{eps}$ is chosen. The concept of this method is not to find a general purpose *eps* that is independent of the dataset, but to determine it for each dataset. By expanding the range of candidates of $\hat{eps}$, the proposed method can be applied to various datasets. However, the range must be manually specified.

**Step 1:**  Perform DBSCAN on the training data of one person by giving each of the *Z* *eps* in a specific candidate of *eps* and calculate $R_{outlier}$, $D_{K}$, $|D_{K}|$, and FMI.
**Step 2:**  Exclude *eps* whose $R_{outlier}$ is more than the threshold value ($th_{out}$).
**Step 3:**  Exclude *eps* whose $D_{K}$ is below the threshold ($th_{dk}$).
**Step 4:**  Exclude *eps* whose $|D_{K}|$ is above the threshold ($th_{dkabs}$).
**Step 5:**  Specify the *eps* with the maximum FMI from the remaining set of *eps* as $\hat{eps}$.

In practice, the samples to be clustered are obtained from a particular user of a smartphone. Therefore, we thought that it would be better to use the training data of only one subject to find $\hat{eps}$. In Step 1, DBSCAN is performed on the training data of one subject while using *E*. The clustering result using $e_{i}$ is expressed as $Res(e_{i})$, which is, a set consisting of samples and corresponding clusters. In addition, the evaluation parameters for $Res(e_{i})$ are expressed as $R_{outlier}\left(Res\left(e_{i}\right)\right)$, $D_{K}\left(Res\left(e_{i}\right)\right)$, and $FMI(Res\left(e_{i}\right))$. In [45], a number of samples were judged to be abnormal, and clustering could not be appropriately performed if *eps* was small, i.e., 0.5, as compared to 1.5 or higher. Therefore, Step 2 excludes *eps* that causes a number of samples to be judged as abnormal using $R_{outlier}\left(Res\left(e_{i}\right)\right)$ and the threshold $th_{out}$. In [45], $R_{outlier}$ was approximately 0.00–0.01 when performing DBSCAN with an optimal *eps*. Therefore, an ideal $th_{out}$ should be set between 0.00 and 0.01. Regarding $D_{K}$, a positive value is more desirable than a negative value, as explained in Section 4.1.1. To prevent $D_{K}$ from being negative, Step 3 excludes *eps* with a small number of clusters while using $D_{K}\left(Res\left(e_{i}\right)\right)$ and the threshold $th_{dk}$. Therefore, the ideal value for $th_{dk}$ is 0.0. It is important that $D_{K}$ is close to zero, because the user is burdened with labeling if the number of new position candidates is too large when compared to the ideal number of positions. Thus, Step 4 excludes *eps*, which causes a large number of clusters to avoid this case using $|D_{K}\left(Res\left(e_{i}\right)\right)|$ and the threshold $th_{dkabs}$. Therefore, the ideal value for $th_{dkabs}$ is a number slightly greater than 0.0. Let *H* be a subset of *E* remaining up to Step 4. Finally, in Step 5, using an evaluation parameter that represents the similarity between the clustering results and the correct labels, i.e., FMI, the *eps* that has the largest FMI is chosen from *H* as $\hat{eps}$.

In the proposed method, all of the *eps* in *E* could be excluded by Step 5 in the worst case. Therefore, we considered it to be effective for making $th_{dk}$ a little smaller and $th_{out}$ and $th_{dkabs}$ a little larger than the ideal value that is mentioned above. Section 4.2.1 shows the thresholds used in this article.

The proposed method can be regarded as a hyperparameter adjustment method in DBSCAN. Grid search, which is one of the most famous hyperparameter adjustment methods, is a method for determining an appropriate value by searching for the value that maximizes the specific accuracy, and is effective for supervised learning [59]. On the contrary, unsupervised learning, such as clustering, cannot generally apply grid search, because there is no correct label at the time of execution. However, because the proposed framework already contains labeled training data, it is possible to calculate the performance when clustering is performed on that data. Taking advantage of this, determining a hyperparameter using training data is the concept of the proposed method. Moreover, in grid search, the value that maximizes the specified accuracy is regarded as the optimum value, but, as mentioned above, there is a special requirement that $D_{K}$ should be positive in the proposed framework. Therefore, we proposed an original hyperparameter adjustment method while using various evaluation parameters.

### 4.2. Experiment: Effectiveness of the Proposed $\hat{eps}$ Search Method

Section 4.2.1 shows the procedure for specifying $\hat{eps}$ using the proposed method, whose results are presented in Section 4.2.4, and the resultant values are used in the experiment presented in Section 4.2.2. Section 4.2.2 presents the procedure for evaluating the performance of DBSCAN with $\hat{eps}$, and Section 4.2.5 shows the results. Section 4.2.3 introduces the datasets used in the experiment. Section 4.3 discusses the effectiveness of the proposed method. From this section onward, it is to be assumed that t-SNE is performed for preprocessing when performing new position candidate identification. Two-dimensional data converted by t-SNE, as shown in Figure 3, Figure 4 and Figure 5, are given to DBSCAN. Additionally, the distance between samples in DBSCAN is calculated while using the Euclidean distance. Python 3.7.4 and the scikit-learn 0.21.3 library were used for the experimental environments in this work.

#### 4.2.1. Searching Optimal eps

The proposed $\hat{eps}$ search method was evaluated by measuring the performance of DBSCAN using a set of values of *eps*. In this section, we applied the proposed $\hat{eps}$ search method in order to specify $\hat{eps}$. Meanwhile, the performance of DBSCAN using the $\hat{eps}$ specified in this section was measured in Section 4.2.2.

**Figure 8:**
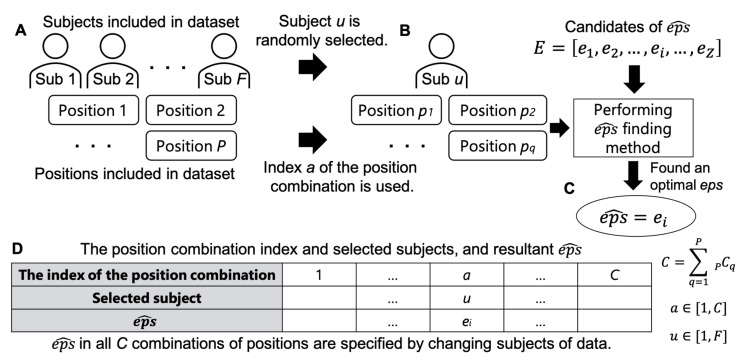
Procedure of the evaluation of finding $\hat{eps}$.

The data used for $\hat{eps}$ searching may contain multiple positions. Therefore, the search method was performed by providing all possible position combinations. For example, if a dataset includes *P* positions, the number of possible combinations of positions is $C=\sum_{q=1}^{P}{}_{P}C_{q}$, which means that the total number of *eps* obtained from the dataset is *C*. Figure 8 illustrates the procedure of this experiment. The data of *P* positions from *F* subjects were assumed to be included in the dataset (Figure 8A). A combination of positions was represented as an index (a∈[1,C]) and sequentially specified, in which a combination consisted of *q* positions with varying elements per index. Subsequently, $\hat{eps}$ was searched using the data of the position combination *a* of subject *u* (Figure 8B). In this way, $\hat{eps}$ was chosen from the candidates of *eps* in *E* (Figure 8C). For each position combination, $\hat{eps}$ was found by randomly choosing the subject (*u*) from *F* persons who provided training data. Thus, a table was obtained, as shown in Figure 8D.

In our previous work, we examined the *eps* of a wide range of a small granularity and found an approximate range of $\hat{eps}$ [45]. In this article, we verified whether the clustering results can be improved by narrowing the range of the candidates of *eps* and making the granularity finer. Thus, we specified two sets of *E*, in which we called cases with wide and coarse grain “pattern WC” and the other cases with narrow and fine grain “pattern NF”. Section 4.2.3 shows the concrete values of the candidates of *eps* for each pattern. In addition, $th_{out}$, $th_{dk}$, and $th_{dkabs}$ were set to 0.05, −3.0, and 5.0, respectively.

#### 4.2.2. Evaluation on Clustering Performance Using Optimal eps

The effectiveness of performing DBSCAN with $\hat{eps}$ was investigated. We assumed that the novelty sample pool contained data from a particular set of position combinations, i.e., combination index *b*, when used by subject *v*, and DBSCAN was performed on this condition. Similar to Section 4.2.1, *b* varied from 1 to *C*. We utilized the *eps* that was obtained in the calculation of Section 4.2.1, which represents $\hat{eps}$ for each position combination index (Figure 8D). Ten *eps* with different position combinations were randomly selected from the previous results, in which the values that were obtained from subject *v* were excluded. This is because the provider of the data to be clustered and that of the data to calculate *eps* were the same subject. At the beginning of using the system in practice, no data from the user were included in the training data for the position classifier, which is generally a difficult condition when compared to that of using the user’s data, although the proportion of the user’s data becomes the majority across a long period of time of use. Thus, we evaluated the system under such difficult conditions. Ten clustering results (Res(eps)) were obtained for each position combination (*b*) of a particular subject (*v*) selected randomly from *F* subjects, and the evaluation parameters (Acc, $R_{outlier}$, $D_{K}$, and FMI) are calculated accordingly. The definition of Acc is presented in the next paragraph. Note that the test subject *v* was randomly chosen for each position combination. The averages of these parameters over 10 trials were calculated as resultant values for each combination of positions and, finally, the overall averages of *C* combinations of positions were obtained. $D_{K}$ was compared to those of *X*-means. Moreover, FMI and Acc were compared to those of *k*-means, which was performed by specifying the number of clusters as the ideal number. Additionally, $R_{outlier}$ was compared to that of DBSCAN when *eps* was set to 0.5.

Here, we describe Acc, one of the evaluation parameters. The results of clustering were assumed to be provided by users after asking for the name of new positions, which were then used to retrain the recognition model for use as training data, as described in Section 3. Hence, it is important to be able to recognize the test data correctly after retraining. Therefore, the accuracy of the recognition after retraining (Acc) is defined by Equation (4), given that the number of correctly classified samples of the test data was $N_{correct}$ and the number of all samples of the test data given to the recognition model was $N_{test}$.

(4) $$\begin{array}{c} Acc=\frac{N_{correct}}{N_{test}} \end{array}$$

**Figure 9:**
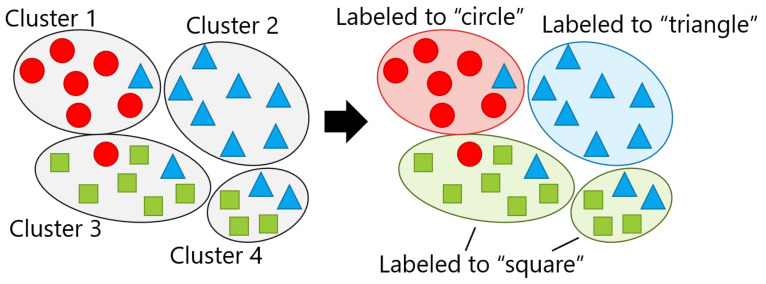
Labeling: the left and right parts are the results of clustering and simulated labeling, respectively.

Retraining requires labeling the identified clusters by the user in advance; however, in this article, labeling was conducted by a simulated user, which means that each identified cluster was marked with the label that was assigned to the largest number of the constituent samples while assuming that the user correctly labels it. Figure 9 presents an example where the samples with one of three labels, i.e., circle, triangle, or square, are clustered at k=4. At this time, it was assumed that each of the four identified clusters contained a mixture of the three labels, as shown on the left side of the figure. Thus, each cluster was labeled by the most common label, as shown on the right side of the figure, in which "square" appeared in two clusters. The smartphone user’s own test data were given to the re-trained classification model, and then Acc was calculated. In this article, using the RandomForest classifier [60] as the recognition model, Acc was calculated by 10-fold cross-validation.

#### 4.2.3. Datasets

Three datasets [4,20,23] were used in the experiments, which consisted of three-axis acceleration signals that were obtained from a wide variety of possible carrying positions (Table 2). SR and wsize refer to the sampling rate of the row data and the window size to cut out to calculate the features, respectively. All of the data were provided by the volunteer subjects during walking. Feature vectors were calculated based on the raw data signal, which consisted of 30, 63, and 16 types of feature that originated from both the time and the frequency domains for datasets A, B, and C, respectively. In dataset A, the correlation coefficients and six common statistical indicators (i.e., mean, variance, maximum value, minimum value, skewness, and kurtosis) were used as features. The features from each original work were used in datasets B and C.

As shown in the table, 11, nine, and four positions were included in the datasets A, B, and C, respectively. Therefore, for each of the three datasets (A, B, and C), the number of possible combinations of positions (*C*) were 2047 ($=\sum_{q=1}^{11}{}_{11}C_{q}$), 511 ($=\sum_{q=1}^{9}{}_{9}C_{q}$), and 15 ($=\sum_{q=1}^{4}{}_{4}C_{q}$), respectively.

The elements of the set of candidates of $\hat{eps}$ (*E*), i.e., 1.5, 2.5, 5.0, 7.5, 10.0, 12.5, and 15.0, were used in common for all three datasets in pattern WC. On the contrary, in pattern NF, the candidates of $\hat{eps}$ that were centered on the range found in [45] were used for each dataset, which is shown in the rightmost column of Table 2.

#### 4.2.4. Analysis of Searching Optimal *eps*

**Figure 10:**
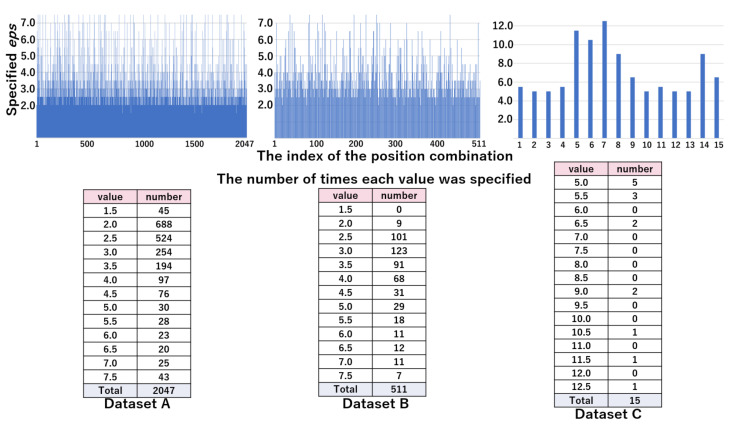
The values of $\hat{eps}$ (top figure) and their distribution (bottom table) in the WC pattern: $\hat{eps}$ candidates are 1.5, 2.5, 5.0, 7.5, 10.0, 12.5, and 15.0.

**Figure 11:**
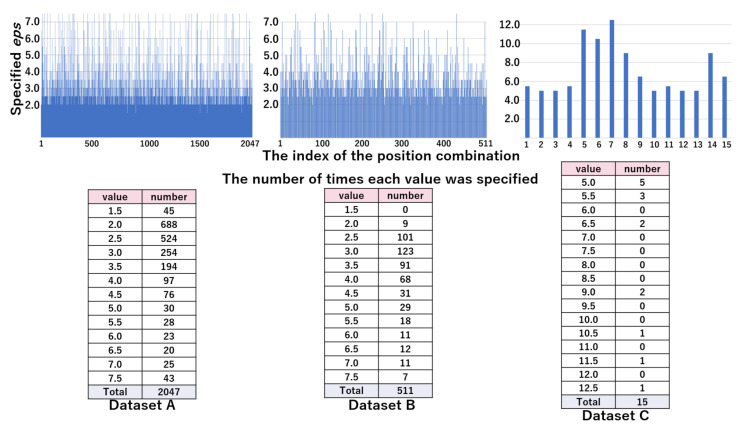
The values of $\hat{eps}$ (top figure) and their distribution (bottom table) in the NF pattern: $\hat{eps}$ candidates are 1.5, 2.0, 2.5, 3.0, 3.5, 4.0, 4.5, 5.0, 5.5, 6.0, 6.5, 7.0, and 7.5 in dataset A and B. $\hat{eps}$ candidates are 5.0, 5.5, 6.0, 6.5, 7.0, 7.5, 8.0, 8.5, 9.0, 9.5, 10.0, 10.5, 11.0, 11.5, 12.0, and 12.5 in dataset C.

The top part of Figure 10 represents the values of $\hat{eps}$ by the position combination indices in the WC pattern. The bottom part of Figure 10 shows the distribution of $\hat{eps}$ by the value of *eps*, which was obtained from the top part. From these results, we can see that the majority of combinations are specified as 2.5 or 5.0 in datasets A and B, and 7.5 in dataset C. Figure 11 shows the values of $\hat{eps}$ by position combination indices and the distribution of $\hat{eps}$ by the value of *eps* in pattern WC. The distribution of $\hat{eps}$ was scattered, due to the increased granularity. In the next section, we evaluated whether the values of $\hat{eps}$ were appropriate.

#### 4.2.5. Results of the Clustering Performance

Table 3, Table 4 and Table 5 summarize the results, showing the evaluation parameters for various comparison conditions. First, the $R_{outlier}$ of DBSCAN with $\hat{eps}$ that was obtained by the proposed method ranged from 0.001 to 0.007, i.e., the columns of the WC and NF patterns. By contrast, an *eps* of 0.5, a default value of *eps* in the DBSCAN class of the scikit-learn library resulted in quite a large $R_{outlier}$ value (larger than 0.9). This means that almost all of the samples were judged as anomalies, and the new position candidate identification was not performed correctly. Next, the $D_{K}$ of DBSCAN with $\hat{eps}$ by the proposed method was close to 0.0, ranging from −0.54 to 1.50, although that of the *X*-means ranged from 3.37 to 6.54. Therefore, it can be confirmed that the proposed *eps* search method contributed to performing the clustering close to the ideal number of positions. Finally, the FMI and Acc of DBSCAN with $\hat{eps}$ by the proposed method were comparable to those of the *k*-means.

### 4.3. Discussion

DBSCAN with the proposed *eps* search method was able to perform clustering with the number of clusters being close to the ideal number of positions as compared to *X*-means. Moreover, FMI, which is an evaluation parameter that shows the performance of clustering, and Acc, which is the most important evaluation parameter in practical use, were comparable to those of *k*-means, in which the number of clusters was given as the ideal number. When a user starts using the smartphone terminal, the training data of others already exist. DBSCAN with the proposed method can considered an effective clustering method for the new position candidate identification in the proposed framework while considering that only these data were used and that the user did not need to adjust any parameters.

Regarding the pattern of the range and the granularity of the $\hat{eps}$ candidates, i.e., WC (wide range and coarse grain) and NF (narrow range and fine grain), the values $D_{K}$, FMI, and Acc of the NF pattern were slightly better than those of the WC pattern, where the tick of *eps* of NF was smaller than that of WC. Thus, the smaller tick of *eps* could be tested, and a closer value to the best value was found, which was a trade-off between the accuracy of the search and computational cost.

We also considered the results of $\hat{eps}$ that were found by the proposed method. We calculated the correlation coefficient between the number of positions and values of $\hat{eps}$. Table 6 summarizes the results. Generally, when the absolute value of the correlation coefficient is less than 0.3, it is considered that there is no correlation. Because the correlation coefficient was less than 0.3 in all three datasets of both patterns, it was assumed that there was no correlation between the number of positions and the value of $\hat{eps}$. Therefore, we considered that the proposed method has the potential to find an optimal *eps*, regardless of the number of positions of the training data used.

In our previous work, the values of $\hat{eps}$ were 2.5–5.0 for datasets A and B, and approximately 7.5–10.0 for dataset C [45]. From the lower tables shown in Figure 10, we investigated whether the proposed search method could find the optimal *eps*. The rate of times identified as $\hat{eps}$ was 0.93 (=(1443+456)/2047), 0.88 (=(180+269)/511), and 0.47 (=(6+1)/15) for datasets A, B and C, respectively, which shows the number of times identified as $\hat{eps}$ was small in dataset C. Nevertheless, Acc and FMI of DBSCAN using *eps* found by the proposed method were comparable to those of *k*-means in all datasets, as shown in Table 3, Table 4 and Table 5. Therefore, the proposed method was not always able to find the optimal value of *eps*. However, this does not mean that we found significant outliers.

## 5. Timing of New Position Candidate Identification

### 5.1. Overview

New position candidate identification was performed on unknown samples that were detected by novelty detection and stored in the novelty sample pool, as shown in Section 3. In the experiments of Section 4, all samples in each dataset were given for the new position candidate identification as those judged as unknown samples; however, in practice, the new position candidate identification process should be invoked when a particular state is reached. Therefore, in this section, we clarify the requirements for appropriate timing to perform this process from two perspectives: the required total number of samples in the novelty sample pool (Section 5.2) and the breakdown of samples between clusters, i.e., positions (Section 5.3). Subsequently, the design of new position candidate identification component is finalized in Section 5.4.

### 5.2. Experiment: Impact of the Number of Samples on the New Position Candidate Identification Process Performance

The objective of this experiment was to see whether there was an impact of the number of samples on the performance of the new position candidate identification process and to find a condition in the number of samples.

#### 5.2.1. Method

**Figure 12:**
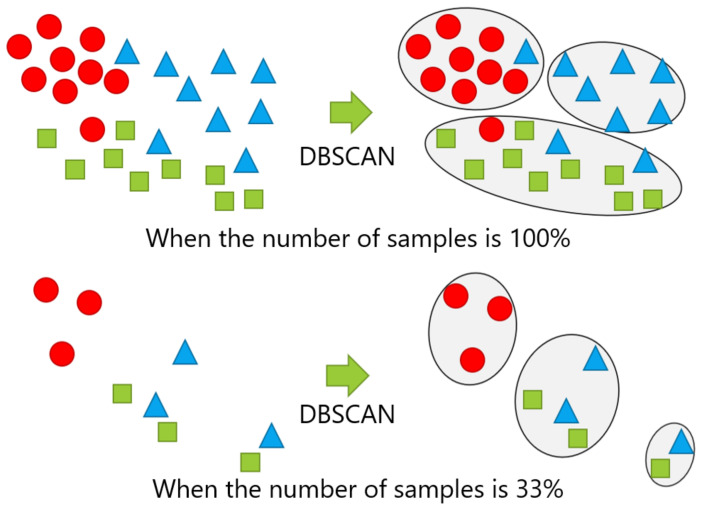
Example of changing the number of samples: The top and bottom are the cases performing DBSCAN with 100% and 33% of the samples, respectively.

The number of samples given for new position candidate identification varied with 100%, 66%, 33%, and 10% of the numbers of the samples in the dataset, which correspond to the entire dataset, two-thirds of the dataset, one-third of the dataset, and one-tenth of the dataset, respectively. The samples were randomly sampled, except for the case with 100% of the dataset. The case with 10% of the dataset represents an extreme case, in which the number of samples was quite small. Figure 12 shows examples of changing the number of samples, in which the cases with 100% and 33% of the samples in each class are shown in the top and the bottom, respectively.

In each of the 2047, 511, and 15 combinations of positions, the data of one subject randomly selected from 70, 20, and 10 subjects were used for the evaluation in datasets A, B, and C, respectively. As evaluation parameters, $D_{K}$, as defined in Section 4.1.1, and Acc, as defined in Section 4.2.2, were calculated for the clustering results. Acc was calculated by 10-fold cross-validation per selected subject. For each evaluation parameter, the average of the total number of combinations of positions was calculated.

Note that it is also possible to change the number of samples itself across the three datasets, such as 50 or 100 samples; however, the appropriate number to form a class may vary, depending on the dataset. In addition, the framework already has samples that make up known classes, in which the number of samples in each class is assumed to be equivalent. Thus, we can specify unified criteria to invoke the new position candidate identification process if no difference is found among the datasets, which is based on the judgement of the relative number of samples to the total number of samples in each class, rather than a unified number.

#### 5.2.2. Results

Table 7 and Table 8 show Acc and $D_{K}$, respectively. At 10%, Acc dropped significantly and $D_{K}$ also became a significantly large negative number. Thus, when the number of samples is extremely small as compared to the number of samples of training data, new positioncandidate identification should not be performed. On the contrary, when the number of samples given was reduced from 100% to 66% or 33%, Acc decreased, but the degree of decrease was small compared to the case between 33% and 10%—especially for datasets A and C, even Acc at 33% was 0.90.

$D_{K}$ tended to be negative as the number of samples given was smaller. It is desirable that $D_{K}$ be positive rather than negative, as mentioned in Section 4.1.1. Although the values of the case with 100% and 66% in dataset C was negative, the absolute value was quite small when compared to the case with 10%. Therefore, as long as the number of samples to be given is not extremely small, such as 10%, the new position candidate identification process can be performed at any time.

### 5.3. Experiment: Impact of the Breakdown of Positions on the New Position Candidate Identification Process Performance

The evaluation presented in Section 5.2 considered the total number of samples in the novelty sample pool, in which each position was assumed to be included in equal proportions. For example, in the case of 66%, Position-1, Position-2, ..., and Position-*n* were equally chosen 66% of time from the dataset. However, in practice, the breakdown of positions from which the samples in the novelty sample pool are obtained is not necessarily equal. Therefore, another evaluation was performed with a different breakdown of samples between positions, such as 66% of the samples of Position-1 and 33% of the samples of Position-2.

#### 5.3.1. Method

**Figure 13:**
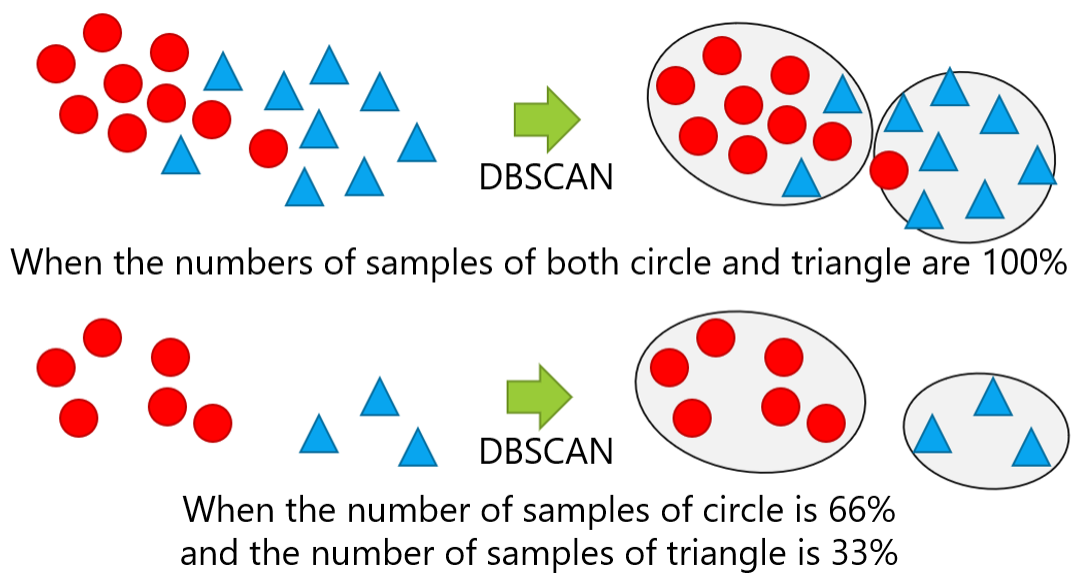
Example of changing the breakdown of positions: The top and bottom are the cases with 100% of both the circle and triangle classes and with 66% of the circle and 33% of the triangle classes, respectively.

For each position, the number of samples given for the new position candidate identification was changed from four levels of ratio: 100%, 66%, 33%, and 10%. The samples were randomly sampled, except for the case with 100% of the dataset, as per in the evaluation of Section 5.2. Figure 13 shows examples of changing the breakdown of positions, in which the top of the figure indicates the case with 100% of the number of both circle and triangle classes, while the bottom shows the case, where 66% of the circle class and 33% of the triangle class were used.

The number of combinations increased exponentially as the number of positions increased, because there were ${}_{4}H{}_{n}$ combinations of the breakdowns to be verified at *n* positions. In addition, in the experiments in Section 4.2 and Section 5.2, the evaluations were performed while using all combinations of positions; however, it is unlikely that one user will have a smartphone in three or more new positions in a short period of time in practice. Therefore, from the viewpoint of experimental cost and practicality, only the case where the number of positions is two was verified in this evaluation, which limits the combination of the levels of proportions to 10 ($={}_{4}H_{2}$).

In each of the 55 ($={}_{11}C_{2}$), 36 ($={}_{9}C_{2}$), and 6 ($={}_{4}C_{2}$) combinations of positions, the data of one randomly selected subject from 70, 20, and 10 subjects were used for the evaluation in datasets A, B, and C, respectively. Similar to the evaluation shown in Section 5.2, Acc and $D_{K}$ were used as the evaluation parameters. Acc was calculated based on 10-fold cross-validation per selected subject. Each evaluation parameter was calculated as the average of the results of 55, 36, and six combinations of positions in datasets A, B, and C, respectively.

#### 5.3.2. Results

Table 9 and Table 10 show Acc and $D_{K}$, respectively. Let the results when the sample of one position and the sample of the other position are *a*% and *b*%, respectively, be represented as *a*–*b*% (a,b∈{100,66,33,10}). Acc tended to decrease when the number of samples in one or both positions was 10%. Otherwise, Acc was 0.90 or higher in all datasets. Regarding $D_{K}$, the smaller the number of samples given, the larger the negative number it tended to be. Moreover, except for 33–10% and 10–10%, it was within ±1. Note that the results of 100–100%, 66–66%, 33–33%, and 10–10% were obtained in the same situation as that of 100%, 66%, 33%, and 10% of Section 5.2.2, because the number of samples of the two positions was changed at the same ratio; however, the results are slightly different, because the combinations of only two positions (55, 36, and six combinations in datasets A, B, and C, respectively) were tested, while the experiment shown in Section 5.2.2 used all possible combinations in each dataset (2047, 511, and 15 combinations in datasets A, B, and C, respectively).

$D_{K}$ tended to be negative when the number of samples given was small, probably because there were not enough samples to form a cluster (class). On the contrary, if the number of samples was 33% or more at both positions, Acc exceeded 0.90, which suggests that it is not always necessary to use massive samples, such as 100–100%, in order to accurately form the class. Thus, new position candidate identification can be performed when there are more than 33% of samples at each position.

### 5.4. Discussion

Based on the results presented in Section 5.2.2 and Section 5.3.2, we found that it is appropriate to perform new position candidate identification when there are samples of 33% or more at each position relative to samples of the training data. However, there is an issue; the breakdown of the samples collected in the novelty sample pool is unknown in practice. Thus, it is difficult to determine whether or not the above condition is satisfied. Therefore, predicting the breakdown of the samples in the pool is a subject for future work.

We propose another timing standard instead of using a timing standard of performing new position candidate identification when the condition is satisfied, as follows:

- The new position candidate identification process can be periodically invoked to check whether at least 33% of the data for each new class candidate relative to the number of original training data are stored. If the condition is satisfied, the result is adopted; otherwise, the samples remain stored in the novelty sample pool for future use.

**Figure 14:**
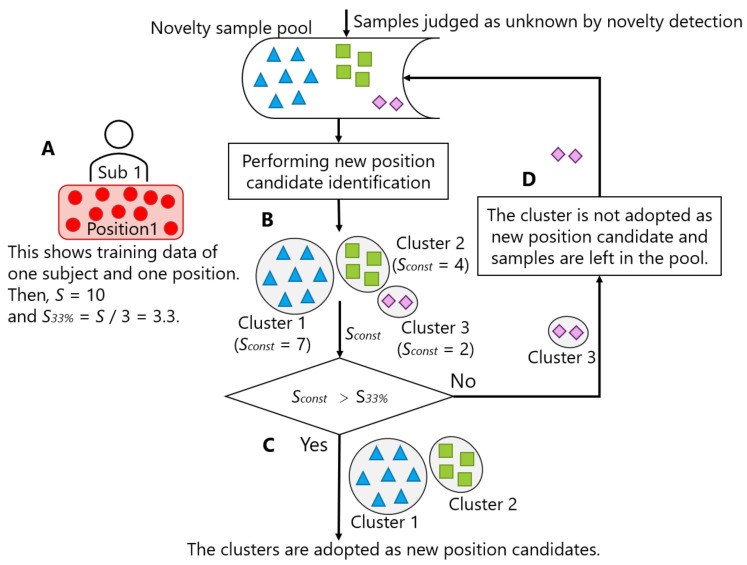
Process of deciding whether to adopt the result of new position candidate identification.

Figure 14 illustrates the process of deciding whether to adopt the result of new position candidate identification. Let *S* be the number of samples per person per position included in the training data (Figure 14A). In the figure, we set *S* to 10. New position candidate identification was regularly performed for the samples accumulated in the novelty sample pool (B). Three clusters were identified in the figure. Among the identified clusters, the clusters whose number of constituent samples ($S_{const}$) exceeded *S* / 3 ($S_{33\%}$) can be regarded as new position candidates (C). In the figure, Cluster 1 and Cluster 2, where $S_{const}$ was 7 and 4, respectively, exceeded $S_{33\%}$ (= 3.3). Thus, these two clusters were adopted as new position candidates. On the contrary, the samples of the unsatisfied clusters were left in the novelty sample pool, and the storing in the pool continued (D). In the figure, the samples of Cluster 3 were left in the novelty sample pool, because $S_{const}$ (= 2) did not exceed 3.3. In other words, the condition for determining whether each identified cluster should be adopted as a new position candidate was $S_{const}>S_{33\%}$.

Here, taking too much time to satisfy the condition is a problem, because it slows the addition of the new position to the group of known positions. On the contrary, it is also a problem if the condition is easily satisfied because the position where the user carries the smartphone just for a moment is added to the group of known positions. Therefore, the time to satisfy the timing conditions ($T_{satisfy}$) is estimated, which is defined by Equations (5)–(8). Table 11 summarizes the symbols used in the formulas and the associated values in the datasets. Note that SR and wsize are already presented in Table 2. The estimated $T_{satisfy}$ is presented in the bottom row of the table. Here, the term sample indicates the window to calculate a feature vector for novelty detection and classification.

(5) $$\begin{array}{c} T_{sample}=\frac{wsize}{SR} \end{array}$$

(6) $$\begin{array}{c} S=\frac{S_{all}}{F\times P} \end{array}$$

(7) $$\begin{array}{c} S_{33\%}=\frac{S}{3} \end{array}$$

(8) $$\begin{array}{c} T_{satisfy}=T_{sample}\times S_{33\%} \end{array}$$

It takes 5–12 min. to collect the samples for one cluster to be adopted as a new position candidate, as shown in this table. We considered that the length of time is appropriate because it is long enough to discriminate a position to be added to a group of recognition targets from temporary positions that can be discarded, and short enough to be introduced into the real setting. In addition, we assumed that the labeling of the new position by the user is not performed so often in a day, but it is rather performed approximately once a day, as described in Section 3.1. Therefore, we considered that the above-mentioned rule can take sufficient time to collect the quantity of samples that satisfy the condition of $S_{const}>S_{33\%}$.

We consider it difficult to apply CILF to our application domain, i.e., using data of known class in a real time manner, although CILF shares the idea of detecting new class and updating the recognition model based on the new class information, as described in Section 3.1. This is because CILF assumes a relatively large size of window to perform clustering within the window, which requires a certain amount of time. If the window of data stored in the CILF is made smaller, and the frequency of novelty detection is increased, the amount of data in the window is reduced accordingly. Acc were low when the number of samples given to clustering is extremely small, as shown in the column of 10% in Table 7 and the columns of *a*%–10% (a∈{100,66,33,10}) in Table 9. In addition, as shown in the column of 10% in Table 8 and the columns of *a*%–10% (a∈{100,66,33,10}) in Table 10, $D_{K}$ tended to be negative, and the number of generated clusters was inappropriate. If the amount of data given to the cluster identification function are not large enough due to the reduction of the size of the window in the CILF toward real time processing, the cluster might not be identified properly as described above. We showed the criteria for an appropriate number of samples for cluster identification through the experiment in this Section. Because the cluster identification is assumed to be performed after a specific amount of samples derived from the criteria are accumulated in the novelty sample pool, the problems that can occur with CILF do not occur with our framework. Thus, we consider that the superiority of our framework to CILF can be claimed.

## 6. Conclusions and Future Work

In this article, we empirically clarified the design principle of a functionality that identifies the candidates of new storing positions of a smartphone toward an extensible personalized on-body device localization system. The role of the functionality is to identify a set of samples that are derived from a single storing position. Thus, clustering is regarded as a key component for preparing a reliable dataset for re-training the classifier and reducing the burden of the user who labels the clusters. Based on a preliminary experiment in our earlier work, we decided to use DBSCAN as a clustering method that does not require the number of clusters, unlike *k*-means clustering. Moreover, DBSCAN seems to be suitable for the distribution of classes in the prospective datasets, i.e., non-spherical, performs anomaly sample detection simultaneously, and it is relatively lightweight when compared to the other clustering methods that do not require the number of clusters, such as affinity propagation and spectral clustering. Thus, we focused on investigating the method of finding an optimal parameter value that controls the granularity of clusters, i.e., Eps-neighborhood or *eps*. Our proposed method is categorized as hyperparameter tuning using a grid search technique, in which the most suitable one is chosen from a number of candidates based on a heuristically built rule. The resultant rule is the answer to Q1 in Section 3.2. The method was evaluated in terms of: (1) the correctness of the estimated number of clusters ($R_{outlier}$ and $D_{K}$), (2) the purity of resultant clusters (*FMI*), and (3) the accuracy of the re-trained classifier (*Acc*) by using three datasets that consisted of different storing positions. The results showed the effectiveness of the proposed method, as follows:

- When compared to a method that estimates the number of clusters (i.e., *X*-means), the proposed method performed clustering closer to the ideal number of positions.
- The proposed method showed a comparable level of *FMI* and *Acc*, as compared to a method that specifies the number of clusters in advance (i.e., *k*-means).

These results indicate that the proposed method of searching for an optimal *eps* contributes to accurately determining clusters in which each sample in a novelty sample pool, i.e., a pool of feature vectors calculated from raw sensor signals that are derived from a possibly unknown position, belongs as if the number of new positions were known in advance. Because the number of clusters is unknown, this offers a great potential to realize an extensible personalized on-body device localization system.

Furthermore, we investigated the timing of invoking the new position candidate identification process, in which two parameters were changed to see the impact on the clustering results: the number of samples in the novelty sample pool and the breakdown of the samples of new class candidates. The findings are summarized as follows:

- The identification process can be invoked if at least 33% of the data for each new class candidate relative to the number of original training data are stored.

However, the findings cannot be directly implemented in the system, because the number of new class candidates is not known. Thus, instead of invoking the identification process after satisfying the condition derived from the findings, the new position candidate identification process can be periodically invoked in order to check whether the above condition is satisfied. In case that the condition is satisfied, the identified clusters can be presented to the user to ask for labeling. Such a periodic evaluation of the condition is the answer to Q2 in Section 3.2. The estimated minimum time to collect the samples required for one cluster to be adopted as a new position candidate, i.e., 33% of original training data, ranged from 5–12 min. in the three datasets. Furthermore, by taking into account a scenario in which labeling by the user is not performed very often (i.e., assuming once a day), we considered that a sufficient number of data could be collected before labeling.

In this article, we designed and evaluated the system while using the datasets that were obtained during walking and, thus, the performance when the users are engaged in other activities is unknown. To understand the versatility of the system, we need to carry out an evaluation using datasets that include various activities. Furthermore, labeling the name of a new class by the user was assumed to be 100% accurate in the experiment; however, in a practical situation where the user makes an annotation before going to bed, for example, he/she might make a mistake. Such mislabeling would contaminate the training data of the recognizers. In [61], the combination of contextual elements, i.e.,time, place, engaged activity, and past answer (label), proved to be effective in recalling the position of a smartphone. Mislabeling would be decreased by incorporating the mechanism of sensing such contextual information and asking to the user into the framework.

## Figures and Tables

**Table 1:** Examples of on-body device position recognition.

Literature	Position
Kunze et al. [16]	Wrist, head, trouser left pocket, and chest pocket
Shi et al. [5]	Chest pocket, front/back trouser pocket, and hand
Vahdatpour et al. [17]	Upper arm, forearm, waist, shin, thigh, and head
Wiese et al. [18]	Pocket, bag, hand, and away from human
Weenk et al. [19]	Pelvis, sternum, head, shoulder, upper arm, forearm, hand, upper/lower leg, and foot
Shoaib et al. [20]	Trouser pocket, arm, wrist, and belt
Coskun et al. [13]	Pocket, bag, and hand
Alanezi et al. [14]	Jacket pocket, front/back trouser pocket, desk, and hand (calling, watching the screen in the portrait direction, and swinging during walking)
Hoseinitabatabaei et al. [21]	Front trouser pocket, shoulder bag, hand, and belt
Diaconita et al. [22]	Pocket, bag, hand, and desk (facing the ceiling and facing the surface of the desk)
Sztyler et al. [15]	Head, chest, upper arm, waist, forearm, thigh, and shin
Fujinami [23]	Neck (hanging), chest pocket, jacket pocket, front/back trouser pocket, and bag (backpack, hand bag, shoulder bag, and messenger bag)
Yang et al. [24]	Jacket pocket, trouser pocket, bag, and hand
Fujinami et al. [4]	Neck (hanging), chest pocket, jacket pocket, front/back trouser pocket, bag (backpack, hand bag, and shoulder bag), and hand (calling, watching the screen in the portrait direction, and swinging during walking)
Shi et al. [25]	Chest pocket, front trouser pocket, jacket pocket, and hand
Hasegawa et al. [26]	Bag, trouser pocket, cushion, towel, rubber, copper, wood, hand, and phone stand
Bieshaar [6]	Jacket pocket, front/back trouser pocket, and backpack
Sang et al. [27]	Arm, hand, and thigh
Chen et al. [28]	Backpack, pocket, bag, and hand
Guo et al. [29]	Backpack, flat, and hand (calling and swinging during walking)
Li et al. [30]	Calf, thigh, upper/lower arm, and back

**Table 2:** The datasets used in the study.

Dataset	# Person	SR	wsize	Position	$\hat{\mathrm{eps}}$ Candidates (Pattern NF)
A [4]	70	50 Hz	256	Neck (hanging), chest pocket, jacket pocket, front/back trouser pocket, bag (backpack, hand bag, and shoulder bag), and hand (calling, watching the screen in the portrait direction, and swinging during walking)	1.5, 2.0, 2.5, 3.0, 3.5, 4.0, 4.5, 5.0, 5.5, 6.0, 6.5, 7.0, and 7.5
B [23]	20	25 Hz	256	Neck (hanging), chest pocket, jacket pocket, front/back trouser pocket, and bag (backpack, hand bag, shoulder bag, and messenger bag)	1.5, 2.0, 2.5, 3.0, 3.5, 4.0, 4.5, 5.0, 5.5, 6.0, 6.5, 7.0, and 7.5
C [20]	10	50 Hz	100	Trouser pocket, arm, wrist, and belt	5.0, 5.5, 6.0, 6.5, 7.0, 7.5, 8.0, 8.5, 9.0, 9.5, 10.0, 10.5, 11.0, 11.5, 12.0, and 12.5

**Table 3:** Each evaluation parameter of DBSCAN (dataset A).

	DBSCAN (WC Pattern)	DBSCAN (NF Pattern)	DBSCAN (eps=0.5)	*X*-Means	*k*-Means
$R_{outlier}$	0.004	0.007	0.969	-	-
$D_{K}$	+0.58	+1.10	-	+5.56	-
FMI	0.932	0.935	-	-	0.954
Acc	0.984	0.988	-	-	0.987

**Table 4:** Each evaluation parameter of DBSCAN (dataset B).

	DBSCAN (WC Pattern)	DBSCAN (NF Pattern)	DBSCAN (eps=0.5)	*X*-Means	*k*-Means
$R_{outlier}$	0.002	0.003	0.998	-	-
$D_{K}$	+0.34	+0.54	-	+6.54	-
FMI	0.915	0.922	-	-	0.947
Acc	0.927	0.940	-	-	0.969

**Table 5:** Each evaluation parameter of DBSCAN (dataset C).

	DBSCAN (WC Pattern)	DBSCAN (NF Pattern)	DBSCAN (eps=0.5)	*X*-Means	*k*-Means
$R_{outlier}$	0.001	0.007	0.999	-	-
$D_{K}$	−0.47	−0.54	-	+3.37	-
FMI	0.861	0.865	-	-	0.841
Acc	0.975	0.977	-	-	0.997

**Table 6:** Correlation coefficient between the number of positions and $\hat{eps}$.

	Dataset A	Dataset B	Dataset C
WC pattern	−0.10	−0.20	−0.07
NF pattern	−0.08	−0.14	0.07

**Table 7:** Acc by changing the total number of samples.

	100%	66%	33%	10%
Dataset A	0.99	0.98	0.94	0.35
Dataset B	0.93	0.92	0.82	0.16
Dataset C	0.98	0.98	0.97	0.65

**Table 8:** $D_{K}$ by changing the total number of samples.

	100%	66%	33%	10%
Dataset A	+1.10	+0.19	−1.13	−6.31
Dataset B	+0.54	−0.01	−0.71	−3.70
Dataset C	−0.01	−0.01	+0.07	−0.51

**Table 9:** Acc with various breakdowns of samples.

	100%	100%	100%	100%	66%	66%	66%	33%	33%	10%
	100%	66%	33%	10%	66%	33%	10%	33%	10%	10%
Dataset A	0.99	0.99	0.99	0.93	0.99	0.99	0.81	0.93	0.43	0.01
Dataset B	0.98	0.97	0.96	0.86	0.96	0.95	0.81	0.91	0.23	0.01
Dataset C	0.99	0.99	0.99	0.97	0.99	0.99	0.98	0.99	0.94	0.80

**Table 10:** $D_{K}$ with various breakdowns of samples.

	100%	100%	100%	100%	66%	66%	66%	33%	33%	10%
	100%	66%	33%	10%	66%	33%	10%	33%	10%	10%
Dataset A	+0.52	+0.28	+0.07	−0.31	−0.12	−0.17	−0.88	−0.52	−1.82	−3.08
Dataset B	+0.39	+0.31	+0.13	−0.05	+0.09	−0.01	−0.14	−0.13	−1.58	−2.00
Dataset C	+0.17	+0.08	+0.16	−0.03	+0.17	+0.23	+0.04	+0.08	−0.08	−0.43

**Table 11:** Time to satisfy the timing condition and the parameters for calculation.

	Dataset A	Dataset B	Dataset C
The time per sample ($T_{sample}$)	5.12 s	10.24 s	2 s
Number of persons (*F*)	70	20	10
Number of positions (*P*)	11	9	4
Number of all samples included in dataset ($S_{all}$)	145,661	34,962	22,450
Sample per person per position (*S*)	approx. 189	approx. 194	approx. 561
Sample of 33% ($S_{33\%}$)	approx. 63	approx. 65	approx. 187
Time to satisfy the timing condition ($T_{satisfy}$)	approx. 323 s	approx. 666 s	approx. 374 s

## Data Availability

The data are not publicly available due to continuing study by the authors.
